# Mechanism of Ershen Zhenwu Decoction in ameliorating chronic heart failure via JNK/MAPK-regulated apoptosis: insights from network pharmacology and experimental validation

**DOI:** 10.3389/fcvm.2025.1561963

**Published:** 2025-04-22

**Authors:** Yulong Liu, Xinyue Wang, Maomao Zhang, Dan Cheng, Zhenpeng Zhu, Lan Ge, Xiaoyu Cheng

**Affiliations:** ^1^ First Affiliated Hospital of Anhui University of Chinese Medicine, Anhui University of Chinese Medicine, Hefei, China; ^2^ Xin'an Key Laboratory of Medical Science, Anhui University of Chinese Medicine, Hefei, China

**Keywords:** Ershen Zhenwu decoction, network pharmacology, molecular docking, JNK/MAPK, apoptosis

## Abstract

**Background:**

Chronic heart failure (CHF) is a complex cardiovascular disease caused by different pathological mechanisms. Modern medicine has made advancements in CHF treatment; however, there are still many challenges. Ershen Zhenwu Decoction (ESZWD) is a Xin'an medicine that has been clinically applied for years and had good efficacy against CHF; however, its underlying mechanisms remain undetermined. Therefore, this study aims to investigate the primary molecular mechanisms of ESZWD in CHF treatment and elucidate its multi-target and multi-level mode of action.

**Objective:**

The aim of this study was to investigate the main molecular mechanisms of ESZWD for the treatment of CHF and to elucidate its multi-target and multi-level mode of action.

**Methods:**

This study employed a network pharmacology approach to analyze the main ESZWD components and core targets. Furthermore, primary CHF targets were predicted to develop a protein–protein interaction (PPI) network and perform Gene Ontology (GO) and Kyoto Encyclopedia of Genes and Genomes (KEGG) pathway enrichment analyses. Moreover, molecular docking was carried out to validate the binding between active ingredients and key targets. For *in vitro* studies, myocardial cell injury models were employed, and immunofluorescence, RT-qPCR, Western blot, and flow cytometry were carried out to validate the critical targets of relevant signaling pathways and the specific ESZWD regulatory mechanisms.

**Results:**

Network pharmacology identified 437 targets for 34 major ESZWD components. Of these, 216 drug–disease intersection targets were identified. The PPI network analysis identified the following core targets: STAT3, HSP90AA1, MAPK8, NFKB1, HIF1A, MMP9, PTGS2, BCL2L1, TLR4, and ESR1. GO analysis revealed that these targets were associated with exogenous stimuli responses, phosphorylation regulation, inflammatory response, and protein tyrosine kinase activity. Furthermore, KEGG analysis showed that ESZWD predominantly impacts cancer, inflammatory response, and apoptosis pathways, with c-Jun N-terminal kinase/mitogen-activated protein kinase (JNK/MAPK)-regulated apoptosis being a key pathway. *In vitro* analyses revealed that ESZWD effectively inhibited JNK activation, modulated MAPK signaling, downregulated pro-apoptotic gene expression, and significantly reduced cardiomyocyte apoptosis rates, thus validating the network pharmacology findings.

**Conclusion:**

Our study shows that paeoniflorin, acetylaconitine, and cryptotanshinone bind to key proteins in the JNK/MAPK apoptosis pathway. *In vitro* validation confirms drug serum from ESZWD regulates this pathway, supporting its therapeutic potential for CHF.

## 1 Introduction

Chronic heart failure (CHF) is a complex cardiovascular disease characterized by structural and/or functional abnormalities of the heart, which impair ventricular filling and ejection capacity. It can result from the progression of various cardiovascular diseases to their middle or late stages. Furthermore, CHF has a high mortality rate (1). Epidemiological data indicate that >12.05 million individuals aged ≥25 suffer from CHF in China, and approximately 3 million new cases are reported annually (2). Furthermore, it has been estimated that >26 million people are affected by CHF globally, which poses a significant public health challenge exacerbated by an aging population (3). Although modern medicine can treat CHF, it has certain side effects, economic burdens, and issues related to patient tolerance. As a complementary therapy, Traditional Chinese Medicine (TCM) can effectively improve the patient's quality of life, enhance medication adherence, and mitigate side effects, therefore offering diverse therapeutic options (4).

Ershen Zhenwu Decoction (ESZWD) is a classic prescription from the Cheng family's internal medicine tradition in Xin'an medicine. It comprises the following seven herbs: *Panax ginseng* C.A. Mey. (Ch. name, Hongshen), *Aconitum carmichaelii* Debeaux (Ch. name, Fuzi), *Salvia miltiorrhiza* Bunge (Ch. name, Danshen), *Poria cocos* (Schw.) Wolf (Ch. name, Fuling), *Paeonia lactiflora* Pall. (Ch. name, Baishao), *Atractylodes macrocephala* Koidz. (Ch. name, Baizhu), and *Zingiber officinale* Roscoe (Ch. name, Shengjiang). For over two decades, ESZWD has been clinically applied for CHF treatment. Preliminary studies have revealed that ESZWD can effectively reduce the patient's aldosterone and N-terminal prohormone of brain natriuretic peptide (NT-ProBNP) levels, significantly improving cardiac function and quality of life (5, 6). Furthermore, *in vitro* and *in vivo* analyses confirmed that ESZWD can effectively alleviate oxidative stress and inhibit myocardial fibrosis (7–10). The efficacy of TCM formulations relies on the combination of chemical components within the herbs; however, only those absorbed into the bloodstream exert specific therapeutic effects (11). Our previous study (12) identified 34 active ESZWD components *in vivo* using ultra-high performance liquid chromatography-quadrupole time-of-flight mass spectrometry (UHPLC/Q-TOF-MS) technology. In this study, network pharmacology and molecular docking techniques were carried out to construct a component-core target network to elucidate its modern pharmacological mechanisms. Furthermore, a myocardial cell injury model was employed to validate key targets and signaling pathways. The findings will provide the basis for a detailed investigation of ESZWD's potential mechanisms in CHF treatment to allow the integration of traditional knowledge with contemporary scientific approaches.

## 2 Materials and methods

### 2.1 Acquisition of targets for ESZWD *in vivo* components

Our previous study identified 34 key ESZWD components (12). The chemical structure of these components was retrieved from the PubChem database (https://pubchem.ncbi.nlm.nih.gov/) in SDF format and imported into the SuperPred database (https://prediction.charite.de/) for target prediction analysis.

### 2.2 Identification of disease targets for CHF

The CHF-related targets and their relevant information were assessed using “Chronic Heart Failure” as the keyword in the GeneCards (https://www.genecards.org/) and OMIM databases (https://www.omim.org/). Targets with a relevance score ≥9.2 in GeneCards were selected.

### 2.3 Construction of PPI network and selection of core targets

After merging and removing duplicates, CHF-related gene targets were identified and intersected with the component targets to acquire intersecting genes. A Venn diagram was constructed using the intersecting genes (https://bioinfogp.cnb.csic.es/tools/venny/). For PPI network construction, the intersecting targets were imported into the STRING database (https://cn.string-db.org/), and the species was set as “*Homo sapiens*.” To build the initial PPI model, the minimum interaction confidence threshold was set at “medium confidence (0.400)” (13). To identify core targets within the PPI network was assessed via Cytoscape 3.8.2 and its plugin CentiScaPe 2.2.

### 2.4 GO and KEGG enrichment analysis

The intersecting components and disease targets were imported into the Database for Annotation, Visualization, and Integrated Discovery (DAVID) (https://davidbioinformatics.nih.gov/), and GO and KEGG enrichment analyses were carried out with a screening threshold of *p* < 0.05. The acquired data were ranked by ascending false discovery rate (FDR), and the top 10 GO enrichment results and top 20 KEGG enrichment results were visualized graphically.

### 2.5 Molecular docking

To evaluate ESZWD's regulatory effects on c-Jun N-terminal kinase/mitogen-activated protein kinase (JNK/MAPK)-regulated apoptosis, key targets within this pathway were selected, including JNK, Bcl2, Caspase3, and Bax. The UniProt database (https://www.uniprot.org/) was employed to identify protein receptor information, and their three-dimensional structures were downloaded from the Protein Data Bank (PDB) database (https://www.rcsb.org/). Moreover, the 34 *in vivo* components were imported into the Traditional Chinese Medicine Systems Pharmacology (TCMSP) database (https://www.tcmsp-e.com/), and those meeting the oral bioavailability (OB ≥ 30%) and drug-likeness (DL ≥ 0.18) criteria were filtered out (14). The top three optimal components were selected for molecular docking analysis. The binding potential between these components and the targets was assessed via PyMOL software, and the interactions were visually analyzed using AutoDock Tools 1.5.6.

### 2.6 Preparation of ESZWD

ESZWD comprises the following herbs: processed Fuzi (5 g), Hongshen (6 g), Fuling (10 g), Baizhu (10 g), Baishao (10 g), Danshen (30 g), and Shengjiang (6 g). All herbs were procured from the First Affiliated Hospital of Anhui University of Chinese Medicine (Batch Numbers: 20221201, 211201, 221001, 21120111, 220905, 221223, and 221121, respectively). Herb quality followed the specifications outlined in the 2020 edition of the Pharmacopoeia of the People's Republic of China and the 2022 Anhui Province Chinese Herbal Standards. The herbs were precisely weighed, and Fuzi was first decocted in distilled water (10 times the volume), heated at a high temperature for 30 min, and simmered at low heat for 1 h. Then, the remaining herbs were added and decocted for 30 min, followed by filtration to collect the liquid. The residue was decocted again with distilled water (eight times the volume) for 30 min–1 h on low heat, filtered, and combined with the first decoction. The final mixture was filtered, centrifuged, concentrated, and stored in the refrigerator for later use (9).

### 2.7 Preparation of drug-containing serum

The *in vivo* analyses were authorized by the Animal Ethics Committee of Anhui University of Chinese Medicine (approval number: AHUCM-rats-2024195). Male specific-pathogen-free (SPF)-grade Sprague Dawley (SD) rats (*n* = 12, age = 6–8 weeks, weight = 200–220 g) were acquired from Henan Scibest Biotechnology Co., Ltd. [Animal Qualification Certificate, SCXK (Henan) 2020-0005]. All the rats were housed in the animal facility of the Xin'an Medicine Key Laboratory at Anhui University of Chinese Medicine and categorized into control and drug-containing serum groups (*n* = 6/group). The drug-containing serum group received intragastric administration of 15.84 g/kg of ESZWD twice daily for 1 week, whereas the control group received equal volumes of saline (9, 15). After 1 h of the last administration, blood samples were collected and centrifuged at 12,000 rpm at 4°C to harvest serum, which was then incubated at 56°C for 30 min, filtered through a 0.45 μm filter, and then stored in a refrigerator for subsequent use.

### 2.8 CCK-8 cell viability assay

The viability of AC16 cells (CL-0790, Procell Life Science & Technology Co., Ltd., Wuhan, China) was assessed via the CCK-8 assay (TargetMol, MA, USA) to determine the optimal concentration of drug-containing serum. Briefly, the cells were seeded in 96-well plates for 24 h and then treated with Dulbecco's modified Eagle medium (DMEM) high-glucose medium (G4202-500ML, Servicebio, Wuhan, China) containing 0%, 2.5%, 5%, 10%, 15%, and 20% drug serum. Subsequently, at 12, 24, 48, and 72 h, CCK-8 reagent (10 μl) was added for 1 h. Absorbance was measured at 450 nm using a microplate reader (DR-3506, Huawei Delong, Wuxi, China).

To optimize Ang II stimulation parameters in the myocardial injury model, cells were co-cultured with Ang II (0, 0.5, 1, and 5 μmol/L) for 24 h. Then, at 12, 24, 48, and 72 h, CCK-8 reagent (10 μl) was added for 1 h, and absorbance was measured at 450 nm using a microplate reader. These analyses were carried out to optimize the concentration of drug-containing serum and the Ang II stimulation conditions.

### 2.9 Cell culture and grouping

AC16 cells were cultured in DMEM augmented with 10% fetal bovine serum, 100 U/ml penicillin, and 100 mg/ml streptomycin at 37°C in a 5% CO_2_. At approximately 80% confluency, cells were passaged via trypsin digestion. For experiments, cells were divided into five groups: a blank control group cultured with medium containing 10% blank serum and four treatment groups. The treatment groups were first stimulated with 1 μmol/L Ang II for 24 h and then cultured in media containing 10% blank serum, 10% drug-containing serum, 10% blank serum + SP600125 (10 μmol/L), and 10% drug-containing serum + SP600125 (10 μmol/L), respectively. SP600125 (HY-12041, MedChemExpress, NJ, USA) was employed as a reference inhibitor of the JNK signaling pathway (16).

### 2.10 Immunohistochemistry

AC16 cells on coverslips were preserved using 4% paraformaldehyde (G1101, Servicebio) for 20 min, rinsed with phosphate-buffered saline (PBS) (G0002, Servicebio) thrice, and then incubated with 400 μl of peroxidase-blocking solution (ZLI-9311D, Zhongshan Golden Bridge Biotechnology, Beijing, China) for 25 min at room temperature in the dark. Subsequently, the cells were washed again with PBS three times, blocked with 3% bovine serum albumin (BSA) (4240GR025, Lanjek Science & Technology, Anhui, China) for 30 min at room temperature, and treated overnight with primary antibody (80024-1-RR, Proteintech, IL, USA) diluted to 1:200 in 1% BSA at 4°C. The next day, after three PBS washes, the cells were incubated with a 1:400 dilution of fluorescent secondary antibody (SA00003-2, Proteintech) for 2 h at room temperature in the dark, washed again three times with PBS, and subjected to nuclear counterstaining using DAPI (BL105A, Lanjek Science & Technology) for 10 min in the dark. Finally, the liquid was aspirated, and the coverslips were mounted using an anti-fade mounting medium. The ECLIPSE CI microscope (Nikon, Tokyo, Japan) was employed to capture and analyze the images.

### 2.11 RT-PCR

Total RNA was extracted from AC16 cells using 1 ml of Trizol (BS258A, Lanjek Science & Technology). The RNA was then subjected to chloroform layering, isopropanol precipitation, 75% ethanol washing, and dilution with diethylpyrocarbonate (DEPC) water (HW049401, Sinopharm Group, Shanghai, China). Then, the total RNA was reverse-transcribed into cDNA using a reverse transcription kit (11121ES60, Yeasen, Shanghai, China). The PCR amplification (BL697A, Lanjek Science & Technology) program was as follows: initial denaturation at 95°C for 3 min, followed by 40 cycles of 95°C for 15 s and 60°C for 30 s. The product's specificity was confirmed via the melting curve analysis. Relative gene expression levels were calculated using the ΔΔCq method. Table 1 lists the primer sequences employed.

**Table 1:** Primer sequences for the RT-PCR.

Gene	Sequence (*F*: 5′-3′)	Sequence (*R*: 5′-3′)
GAPDH	ACAACTTTGGTATCGTGGAAGG	GCCATCACGCCACAGTTTC
JNK	TGTGTGGAATCAAGCACCTTC	AGGCGTCATCATAAAACTCGTTC
Caspase-3	GAAATTGTGGAATTGATGCGTGA	CTACAACGATCCCCTCTGAAAAA
BAX	CCCGAGAGGTCTTTTTCCGAG	CCAGCCCATGATGGTTCTGAT
BCL2	GGTGGGGTCATGTGTGTGG	CGGTTCAGGTACTCAGTCATCC

### 2.12 Western blot

The total protein of the cells from each group was acquired and quantified via the bicinchoninic acid (BCA) method (P0009, Beyotime Biotechnology, Shanghai, China). Then, the proteins were mixed with a loading buffer and subjected to electrophoresis at a constant voltage of 90 V in a Mini-Protean Tetra System (Bio-Rad, CA, USA). Proteins were then transferred onto membranes at a constant current of 300 mA for 70 min, blocked with 5% skim milk for 2 h, washed with tris buffered saline with tween 20 (TBST), and incubated overnight at 4°C with primary antibodies diluted in antibody diluent: β-actin (1:5,000, 66009-1-Ig), JNK (1:3,000, 66210-1-Ig), p-JNK (1:1,000, 80024-1-RR), Caspase-3 (1:500, R23727), Bcl-2 (1:200, sc-7382), and Bax (1:1,000, 2772T) (all antibodies from Proteintech, Zenbio, Santa Cruz, or CST). Then, the membranes were probed with horseradish peroxidase (HRP)-conjugated secondary antibodies (ZB-2305, Lanjek Science & Technology) for 1 h at room temperature and washed. Protein bands were visualized using the SH-523 chemiluminescence imaging system (Hangzhou Shenhua Technology, Hangzhou, China), and the band densities were analyzed via ImageJ software. Protein expression levels were normalized to β-actin.

### 2.13 Flow cytometry

The cells (1 × 10^5–6^ in 100 μl/well) were grown in 6-well plates for 24 h at 37°C with 5% CO_2_. Then, the cells were treated and centrifuged at 2,000 rpm for 5 min. Adherent cells were then briefly digested with ethylenediaminetetraacetic acid (EDTA)-free trypsin, collected by centrifugation, and washed twice with PBS (G0002, Servicebio) by centrifugation at 2,000 rpm for 5 min. Finally, 1–5 × 10^5^ cells were resuspended in 500 μl of binding buffer (G0020, Servicebio) and treated with Annexin V-FITC (5 μl) and propidium iodide (PI; 5 μl, BL107B, Lanjek Science & Technology) at room temperature in the dark for 5–15 min. Samples were analyzed within 1 h using a CytoFLEX LX flow cytometer (Beckman Coulter, CA, USA). The analysis settings were: excitation wavelength at 488 nm, Annexin V-FITC detected through the FL1 channel (530 nm), and PI detected through the FL3 channel (≥630 nm). To eliminate spectral overlap and set the gate positions accurately, fluorescence compensation was adjusted using untreated normal cells to distinguish live early apoptotic and late apoptotic/necrotic cells.

### 2.14 Statistical analysis

All statistical analyses were presented as mean ± SEM derived from three independent biological replicate experiments (*n* = 3 biological replicates per group). Two-group comparisons were analyzed using two-tailed unpaired Student's *t*-tests, whereas multiple-group comparisons were evaluated via one-way ANOVA followed by Tukey's *post-hoc* test. A significance level of *p* < 0.05 or *p* < 0.01 was applied. Statistical analyses were performed using SPSS 26.0 and GraphPad Prism 10.

## 3 Results

### 3.1 Prediction of ESZWD targets

The SDF files of the 34 *in vivo* ESZWD components (Supplementary Table S1) were uploaded onto the SuperPred database for systematic target prediction, which identified 437 potential related targets.

### 3.2 Prediction of CHF-related targets

**Figure 1:**
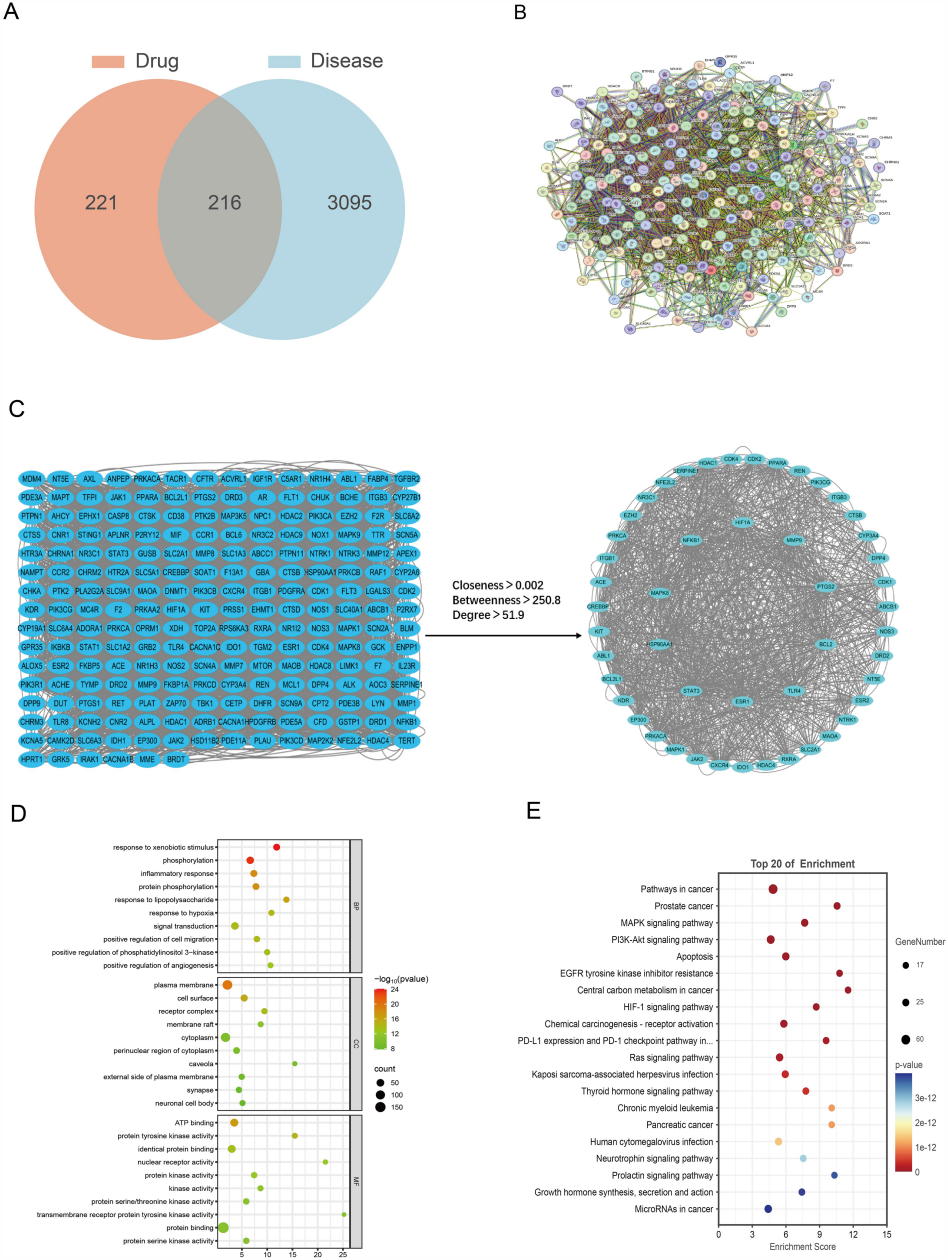
Network pharmacological analysis. **(A)** Venn diagram of drug and disease intersections. **(B)** Protein–protein interaction networks. **(C)** Intersection targets and core targets network. **(D)** GO enrichment analysis results. **(E)** KEGG enrichment analysis results.

The integration of the OMIM and GeneCards datasets identified 3,311 CHF-associated targets. Furthermore, the intersection analysis of the 437 potential ESZWD targets was carried out using the SuperPred database, which revealed 216 genes related to both the drug and the disease (Figure 1A).

### 3.3 PPI network construction

To investigate the mechanism of action of ESZWD, the 216 drug–disease-related targets were imported into the STRING database to construct a PPI network (Figure 1B). Then, Cytoscape was employed to screen the core targets based on thresholds of degree > 51.9, betweenness > 250.8, and closeness > 0.002. The data revealed 49 core targets and 1,158 interaction edges between them. The top 10 key nodes with the highest degree values were STAT3, HSP90AA1, MAPK8, ESR1, NFKB1, HIF1A, MMP9, PTGS2, BCL2L1, and TLR4 (Figure 1C).

### 3.4 GO enrichment analysis

The GO enrichment analysis of the intersecting drug–disease targets was performed using the DAVID, which identified 285 significant GO terms (FDR < 0.01). There were 175 enriched biological processes (BPs), including response to exogenous stimuli phosphorylation, and inflammatory response; 47 enriched molecular functions (MFs), such as plasma membrane-related functions, cell surface, and receptor complex; and 63 enriched cellular components (CCs) including ATP binding, protein tyrosine kinase activity, and identical protein binding (Figure 1D).

### 3.5 KEGG pathway analysis

The DAVID was employed for the KEGG pathway enrichment analysis of the intersecting targets. The data revealed 147 significant signaling pathways (FDR < 0.01), which primarily included cancer, inflammatory responses, and apoptosis-related pathways, such as the MAPK and phosphoinositide-3-kinase–protein kinase B/Akt (PI3K-Akt) signaling pathways, as well as cancer and apoptosis-related pathways (Figure 1E). The results of core target screening were combined to further investigate the mechanism by which ESZWD mediates apoptosis via the MAPK signaling pathway in CHF (17).

### 3.6 Molecular docking

**Figure 2:**
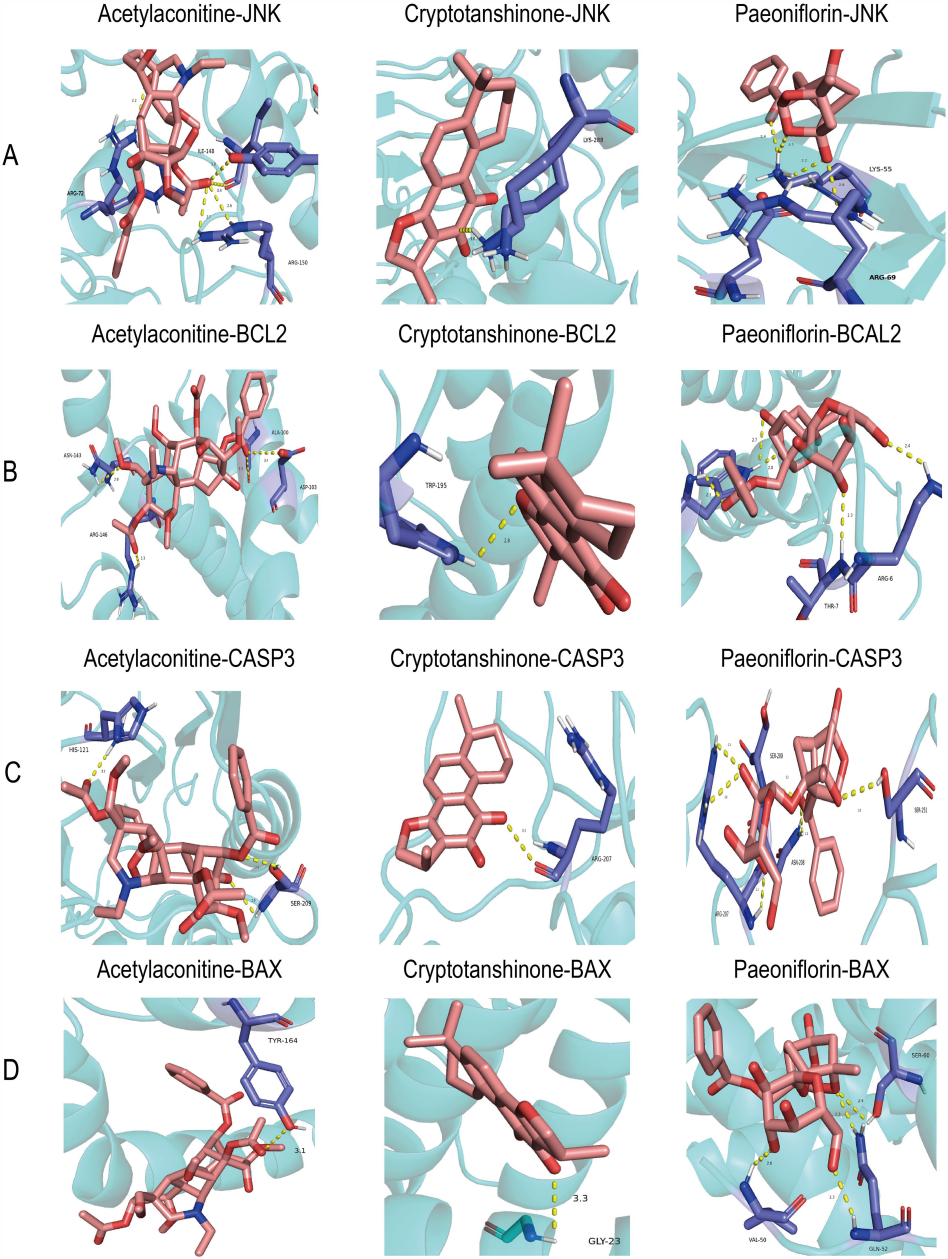
Molecular dockings of the core targets and their corresponding ingredients. **(A)** JNK and acetylaconitine (−7.4 kcal/mol), JNK and cryptotanshinone (−8.8 kcal/mol), JNK and paeoniflorin (−9.7 kcal/mol). **(B)** BCL2 and acetylaconitine (−6.7 kcal/mol), BCL2 and cryptotanshinone (−8.3 kcal/mol), and BCL2 and paeoniflorin (−7.9 kcal/mol). **(C)** CASP3 and acetylaconitine (−7.4 kcal/mol), CASP3 and cryptotanshinone (−8.4 kcal/mol), and CASP3 and paeoniflorin (−8.4 kcal/mol). **(D)** Bax and acetylaconitine (−6.5 kcal/mol), Bax and cryptotanshinone (−6.7 kcal/mol), and Bax and paeoniflorin (−8.3 kcal/mol).

The TCMSP database screening identified three components with high oral bioavailability and excellent drug-likeness: paeoniflorin, acetylhypaconitine, and cryptotanshinone (Table 2). Molecular docking analysis indicated that these compounds form strong and stable hydrogen bonds with key proteins JNK, Bcl2, Caspase3, and Bax. The binding energies of all the proteins with targets were <−0.5 kJ/mol (Table 3). JNK interacted with acetylaconitine via ARG-72, ILE-148, and ARG-150; with cryptotanshinone via LYS-288; and with paeoniflorin via LYS-55 and ARG-69 (Figure 2A). Bcl2 communicated with acetylaconitine through ASN-143, ARG-107, ARG-146, ALA-100, and ASP-103; with cryptotanshinone via TRP-195; and with paeoniflorin via TRP-195, THR-7, and ARG-6 (Figure 2B). Caspase3 formed bonds with acetylaconitine at SER-209 and HIS-121; with cryptotanshinone at ARG-207; and with paeoniflorin at ARG-207, SER-209, ASN-208, and SER-251 (Figure 2C). Bax interacted with acetylaconitine via TYR-164; with cryptotanshinone via GLY-23; and with paeoniflorin via VAL-50, GLN-52, and SER-60 (Figure 2D).

**Table 2:** Optimal *in vivo* components selected based on OB and DL.

Ingredient	Mol	OB	DL
Paeoniflorin	MOL001924	53.87	0.78
Acetylaconitine	MOL004749	37.05	0.19
Cryptotanshinone	MOL007088	52.34	0.39

**Table 3:** Target and corresponding components with molecular docking binding energy.

Ligand component	PubChem CID	Receptor protein	PDB ID	Binding energy (kJ/mol)
Acetylaconitine	21599000	Bcl2	1G5M	−6.7
Acetylaconitine	21599000	Casp3	1GFW	−7.4
Acetylaconitine	21599000	BAX	4BD6	−6.1
Acetylaconitine	21599000	JNK	4L7F	−7.4
Cryptotanshinone	160254	Bcl2	1G5M	−8.3
Cryptotanshinone	160254	Casp3	1GFW	−8.4
Cryptotanshinone	160254	BAX	4BD6	−9
Cryptotanshinone	160254	JNK	4L7F	−8.8
Paeoniflorin	442534	Bcl2	1G5M	−7.9
Paeoniflorin	442534	Casp3	1GFW	−8.4
Paeoniflorin	442534	BAX	4BD6	−6.5
Paeoniflorin	442534	JNK	4L7F	−9.7

### 3.7 The cell viability

**Figure 3:**
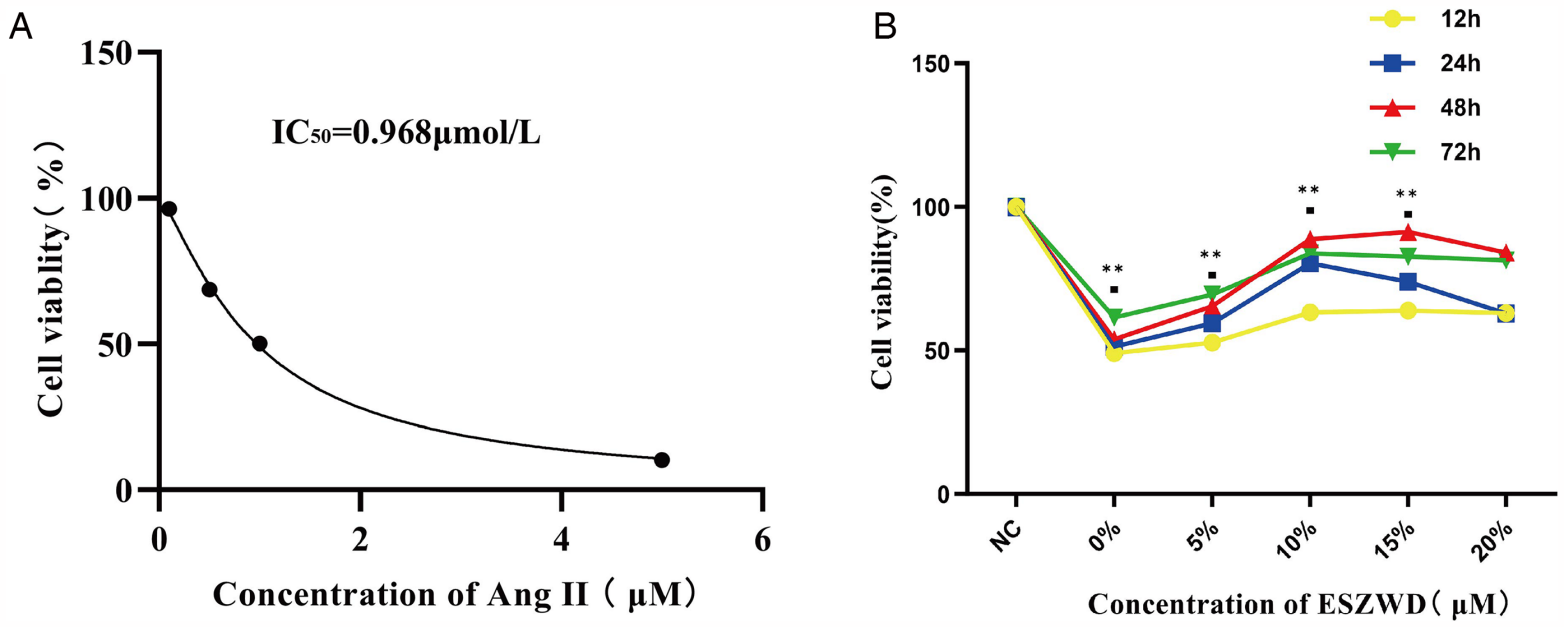
Optimal experimental concentrations of ESZWD and Ang II. **(A)** CCK8 assay for determining the IC50 of Ang II. **(B)** CCK8 assay for determining the optimal concentration of ESZWD. Data are mean ± SD from three independent experiments (*n* = 3 per group). **p < 0.01 when compared with the negative control group.

The optimal Ang II concentrations for stimulating cardiomyocytes and ESZWD drug-containing serum were assessed via the CCK-8 assay. First, the effects of different Ang II concentrations (0, 0.5, 1, and 5 μM) on cell viability were evaluated, which revealed a substantial decrease in cell viability with increasing Ang II concentration. The curve fitting analysis yielded an IC_50_ value of 0.968 μmol/L for Ang II; therefore, the concentration of 1 μmol/L was selected for subsequent experiments (Figure 3A). Furthermore, the impact of varying ESZWD concentrations (0%, 5%, 10%, 15%, and 20%) on cell viability at 12, 24, 48, and 72 h post-treatment was also elucidated. The data showed that the cell viability initially decreased with increasing ESZWD concentration but then gradually recovered. The cell viability stabilized at a 10% ESZWD concentration at 48 h; thus, the 10% ESZWD concentration for 48 h was selected as the interference condition. Moreover, based on the literature review, the 10 μM concentration was selected for the JNK inhibitor SP600125 for subsequent experiments (Figure 3B).

### 3.7 Immunofluorescence test results

**Figure 4:**
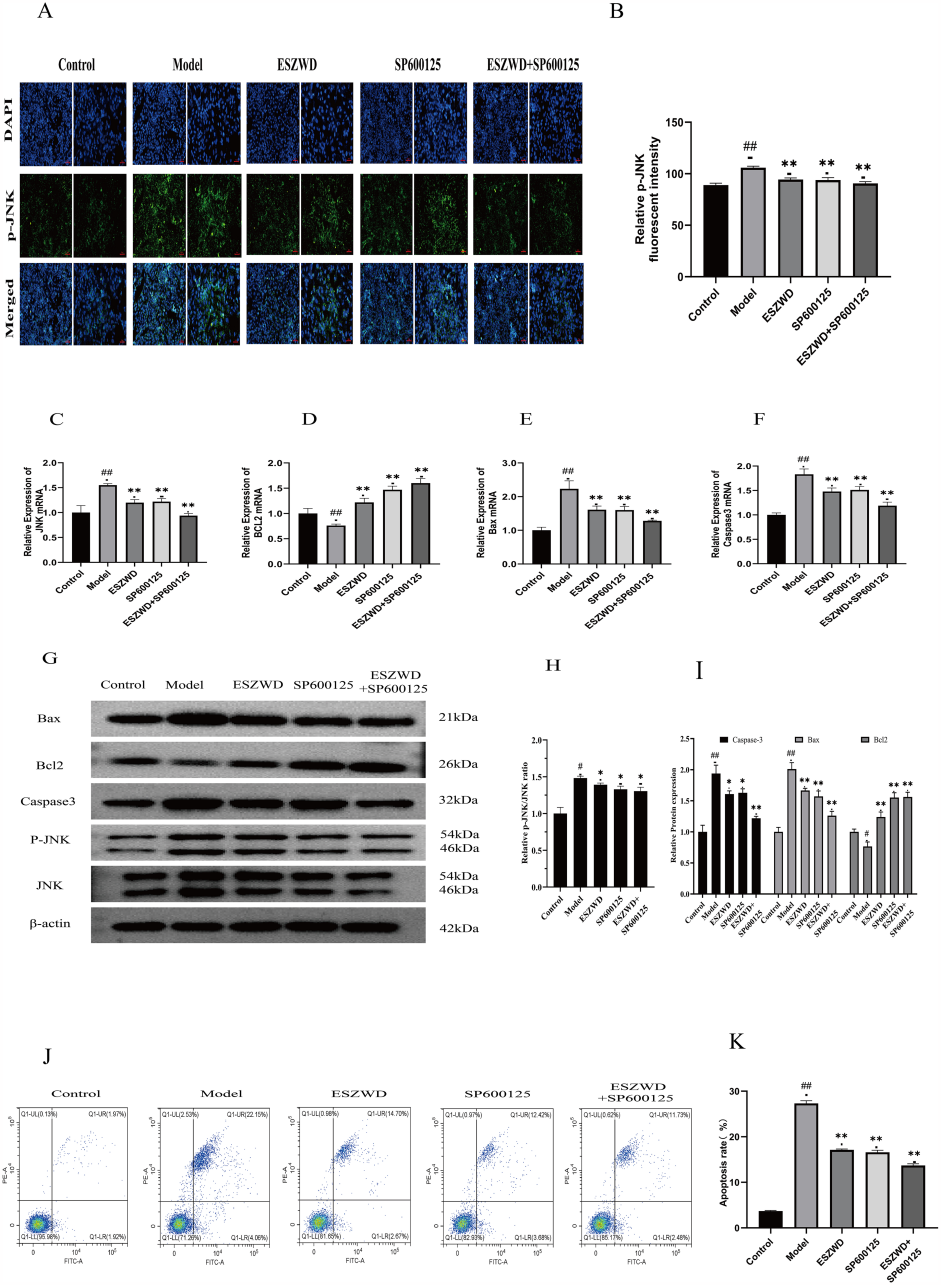
*In vitro* effects of ESZWD on the JNK/MAPK-regulated apoptotic pathway. **(A)** Immunofluorescence detection of p-JNK Expression. **(B)** Quantitative analysis of relative p-JNK fluorescent intensity. **(C–F)** RT-PCR detection results. **(G)** Western blot analysis of Bax, Bcl2, Caspase3, p-JNK, and JNK in different treatment groups. **(H)** The impact of ESZWD on the p-JNK/JNK ratio. **(I)** Quantitative analysis of relative protein expression levels. **(J)** Flow cytometry analysis of cell apoptosis. **(K)** Quantitative analysis of apoptosis rates. Data are mean ± SD from three independent experiments (*n* = 3 per group). *p < 0.05; ** *p* < 0.01 when compared with the model group; ##*p* < 0.01 when compared with the control group.

The effect of ESZWD on JNK gene expression was investigated by assessing the phosphorylated JNK (P-JNK) levels in cardiac muscle cells from each group via immunofluorescence analysis. The data revealed that compared to the control group, the model group cardiac cells had markedly increased P-JNK expression (*p* < 0.01). Moreover, after treatment with ESZWD, SP600125, or ESZWD + SP600125, the P-JNK expression in cardiac cells was markedly reduced compared to the model group (*p* < 0.01; Figures 4A,B).

### 3.8 RT-PCR analysis of key mRNA levels

RT-PCR analysis revealed that relative to the control group, the JNK, Caspase3, and Bax mRNA expression levels were significantly increased (*p* < 0.01), while Bcl2 mRNA expression was significantly decreased (*p* < 0.01) in the cardiomyocytes of the model group. However, the ESZWD, SP600125, and ESZWD + SP600125 treatments markedly reduced the mRNA expression levels of JNK, Caspase3, and Bax (*p* < 0.01), whereas significantly increased Bcl2 mRNA expression (*p* < 0.01), compared to the model group (Figures 4C–F).

### 3.9 Western blot to detect the expression of the key proteins

Western blot analysis revealed that, compared to the control group, the protein expression levels of Caspase3, Bax, and the p-JNK/JNK ratio in the cardiomyocytes of the model group significantly increased (*p* < 0.05 or *p* < 0.01), while that of Bcl-2 significantly decreased (*p* < 0.05). However, after treatment with the ESZWD, SP600125, or ESZWD + SP600125, the protein expression levels of Caspase3 and Bax, as well as the p-JNK/JNK ratio, were significantly reduced compared to the model group (*p* < 0.05 or *p* < 0.01), whereas the Bcl-2 protein levels increased significantly (*p* < 0.01; Figures 4G–I).

### 3.10 Flow cytometry analysis of cell apoptosis

Flow cytometry analysis using Annexin V/PI staining revealed significant differences in apoptosis rates among groups. The results showed that relative to the control group (a total apoptosis rate = 3.89%), the model group had a significantly increased apoptosis rate (26.21%; *p* < 0.01). The ESZWD, SP600125, and ESZWD + SP600125 treatments significantly reduced apoptosis rates (*p* < 0.01), where the total reduction in early and late apoptosis rates in the ESZWD group was 14.7% and 2.67%, respectively; in the SP600125 group it was 12.42% and 3.68%, respectively; and in the combined treatment group it was 11.73% and 2.48%, respectively (Figures 4J,K).

## 4 Discussion

CHF can be caused by various factors, such as genetics, environment, and lifestyle. It is a complex disease with a potential polygenic basis and often does not respond optimally to single-target therapies (18). Therefore, multi-target combination treatments have become the preferred approach for CHF treatment. TCM has multi-component and multi-target properties, which offer comprehensive therapeutic effects by alleviating multiple symptoms and regulating body functions at various levels (19). ESZWD is an optimized herbal formula from the ancient Zhen Wu Decoction from the Xin'an region of China. It has been widely used clinically due to its good efficacy. Notably, recent studies have demonstrated that metoprolol, a commonly used *β*-blocker in clinical practice, frequently induces gastrointestinal adverse effects and fatigue/somnolence in patients with heart failure and preserved ejection fraction. In our trials, the combination of ESZWD and metoprolol not only enhanced cardiac function and optimized neuroendocrine profiles but also significantly reduced these adverse effects (20).

UHPLC/Q-TOF-MS analysis has revealed 34 active ESZWD components (12). This study indicated that these components have 437 potential targets and the intersection of these targets with drug targets revealed 216 drug–disease targets. Furthermore, the topological analysis has revealed that the core targets include STAT3, HSP90AA1, MAPK8, NFKB1, HIF1A, MMP9, PTGS2, BCL2L1, TLR4, and MAPK8. In CHF, NFKB1, TLR4, and ESR1 regulate inflammation (21, 22), while STAT3, BCL2L1, and MAPK8 influence cell survival and death (23). Studies have indicated that HIF1A promotes angiogenesis under hypoxia, MMP9 influences cardiac structure by degrading the extracellular matrix, and HSP90AA1 modulates cardiac remodeling (24, 25). The core ESZWD targets modulate various physiological processes including inflammatory response apoptosis and oxidative stress, which helps to effectively alleviate CHF.

The GO enrichment analysis revealed that the potential ESZWD targets are primarily related to processes including cellular response to external stimuli, phosphorylation regulation, inflammatory response, and protein tyrosine kinase activity, indicating its broad cellular mechanisms and comprehensive signaling pathway modulation. Furthermore, KEGG pathway analysis revealed that ESZWD modulates various pathways critical for cardiac health, such as the MAPK pathway, which is crucial in cardiac remodeling and apoptosis. Abnormal activation of the MAPK pathway promotes cardiomyocyte proliferation, hypertrophy, and fibrosis, therefore worsening cardiac function (26). The PI3K-Akt pathway supports heart function by regulating cell proliferation, differentiation, inflammation, and apoptosis (27). Moreover, the hypoxia-inducible factor (HIF)-1 pathway regulates cardiomyocyte metabolism during hypoxia, thus increasing survival by modulating glycolysis and oxidative phosphorylation (28). Overactivation of the epidermal growth factor receptor (EGFR) pathway has been observed to promote myocardial fibrosis and ventricular remodeling, which deteriorates heart structure and function (29). Both intrinsic and extrinsic apoptosis pathways promote cardiomyocyte programmed death (30). These data suggest that ESZWD plays a multifaceted role in mitigating cardiac dysfunction via different signaling pathways. This study identified paeoniflorin, acetylaconitine, and cryptotanshinone as the top three active components in ESZWD based on oral bioavailability and drug-likeness criteria. Paeoniflorin has antioxidant and anti-inflammatory properties and has been observed to improve heart function by reducing oxidative stress, inhibiting inflammatory responses, scavenging free radicals, and mitigating cardiomyocyte damage (31, 32). Acetylaconitine promotes significant cardiotonic effects by increasing myocardial contraction, optimizing energy metabolism, and improving cardiac pumping efficiency. These effects are specifically beneficial for patients with CHF with weakened cardiac function (33). Cryptotanshinone has indicated anti-inflammatory, antioxidant, and anti-fibrotic properties. This compound can effectively alleviate myocardial remodeling and prevent abnormal changes in cardiac tissue caused by prolonged stress or injury (34, 35). Molecular docking studies revealed that these three compounds had excellent binding affinity and formed stable hydrogen bonds with key receptor proteins including JNK, Bcl2, Caspase3, and Bax. This strong binding confirmed their potential role in cellular signaling and indicated their significant therapeutic value in modulating relevant signaling pathways.

The activation of the JNK/MAPK-regulated apoptosis pathway is essential for cardiac cell apoptosis and functional decline. Under different stresses, such as ischemia, oxidative stress, or inflammation, the upstream mitogen-activated protein kinase kinase kinases (MAPKKKs) activate mitogen-activated protein kinase kinases (MAPKKs), which promotes JNK phosphorylation. Activated JNK then phosphorylates c-Jun in the nucleus to alter gene expression or directly regulate mitochondrial proteins to control apoptosis (36, 37). Caspase3 is an executioner protease that is dependent on the activation of initiator caspases to trigger cellular disintegration. Phosphorylated Bax translocates to the mitochondrial membrane, forms pores, and releases cytochrome c, which activates Caspase9 and Caspase3 (38). The activation of the JNK pathway may inhibit the activation of the anti-apoptotic protein Bcl2, thus promoting Bax pore formation and enhancing apoptosis (39).

*In vitro* investigations revealed that ESZWD-containing serum significantly modulates this apoptosis process, validating its multi-target mechanism. Mechanistically, ESZWD inhibited JNK expression to modulate apoptosis and the expression of Bcl2, Caspase3, and Bax, maintaining cellular homeostasis and reducing unnecessary cell death. Overall, these data highlight the significant role of ESZWD in regulating key signaling pathways and provide robust experimental evidence for its potential as a multi-target therapy for CHF.

Although this study demonstrated the positive effects of ESZWD in an *in vitro* model of chronic heart failure, the lack of *in vivo* validation using animal models limits the comprehensive evaluation of its pharmacological effects and safety profile. Future studies should address this gap through animal experiments to establish a more robust experimental foundation for the further development of ESZWD.

## 5 Conclusion

This study demonstrated that key components of ESZWD, paeoniflorin, acetylaconitine, and cryptotanshinone, can bind to critical proteins including JNK, Bcl-2, Caspase-3, and Bax. *In vitro* experiments validated that the drug-containing serum of ESZWD regulates the JNK/MAPK apoptosis pathway, providing a theoretical basis for its application in treating CHF.

## Data Availability

The datasets presented in this study can be found in online repositories. The names of the repository/repositories and accession number(s) can be found in the article/Supplementary Material.
