# Supplementary figures and images for: Mechanism of Ershen Zhenwu Decoction in ameliorating chronic heart failure via JNK/MAPK-regulated apoptosis: insights from network pharmacology and experimental validation
Source: Front Cardiovasc Med. 2025 Apr 22;12:1561963. doi: 10.3389/fcvm.2025.1561963 (PMC12052711; doi:10.3389/fcvm.2025.1561963)

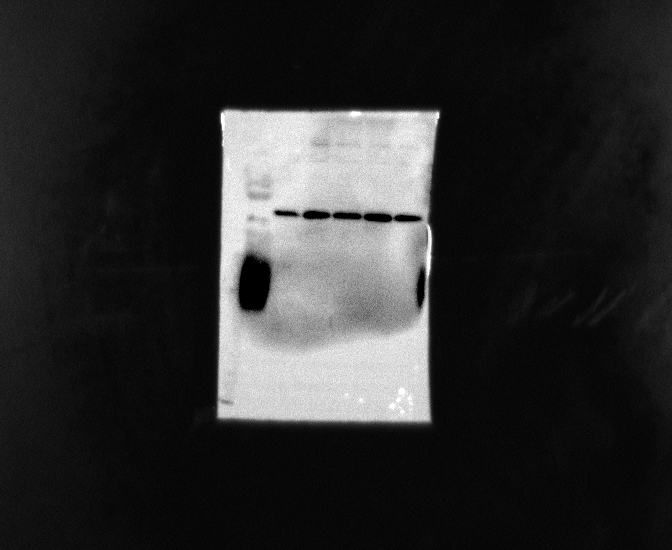

Supplement: Supplementary file 1 [file Datasheet1.zip › Date sheet 1/western blot/Figure 4G/BAX-1.jpg]

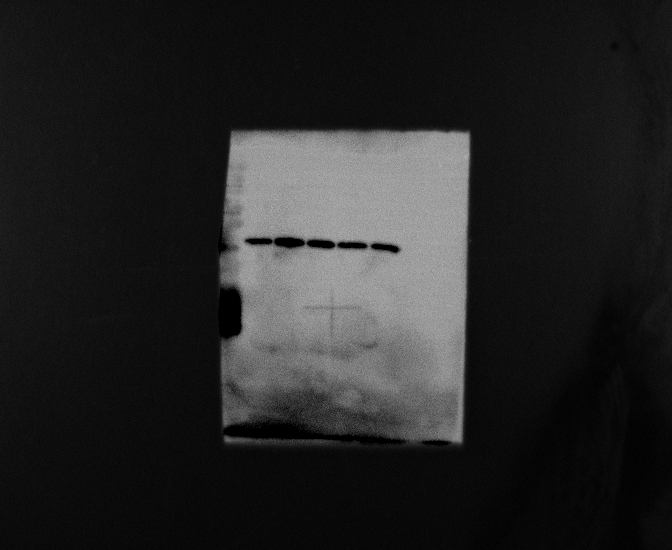

Supplement: Supplementary file 1 [file Datasheet1.zip › Date sheet 1/western blot/Figure 4G/BAX-2.jpg]

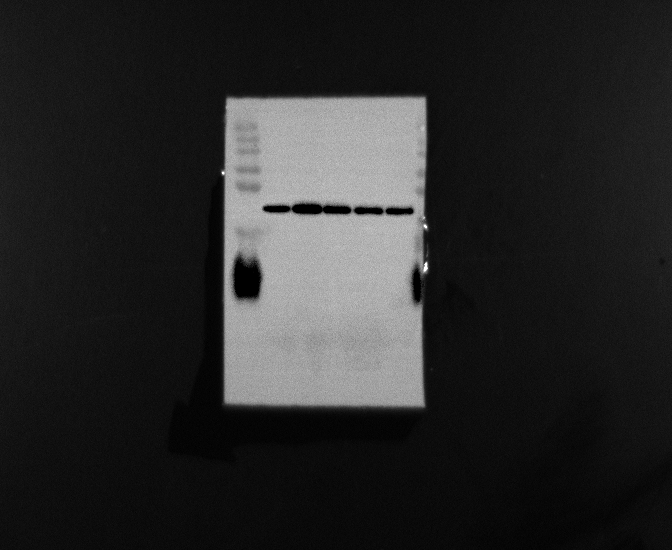

Supplement: Supplementary file 1 [file Datasheet1.zip › Date sheet 1/western blot/Figure 4G/BAX-3.jpg]

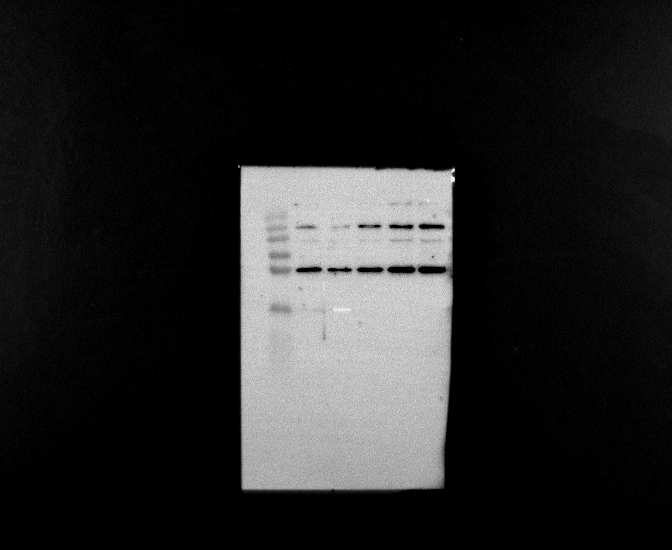

Supplement: Supplementary file 1 [file Datasheet1.zip › Date sheet 1/western blot/Figure 4G/Bcl-2-1.jpg]

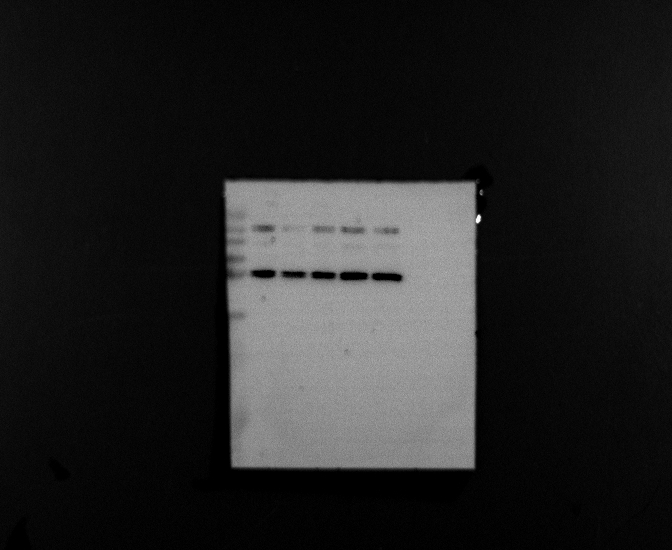

Supplement: Supplementary file 1 [file Datasheet1.zip › Date sheet 1/western blot/Figure 4G/Bcl-2-2.jpg]

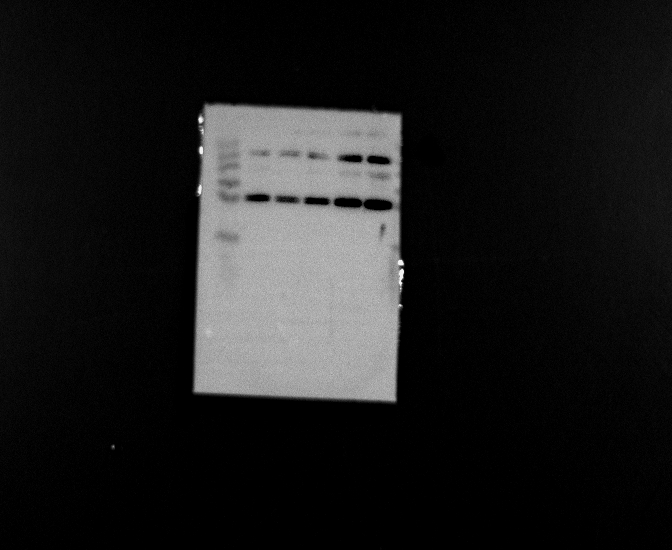

Supplement: Supplementary file 1 [file Datasheet1.zip › Date sheet 1/western blot/Figure 4G/Bcl-2-3.jpg]

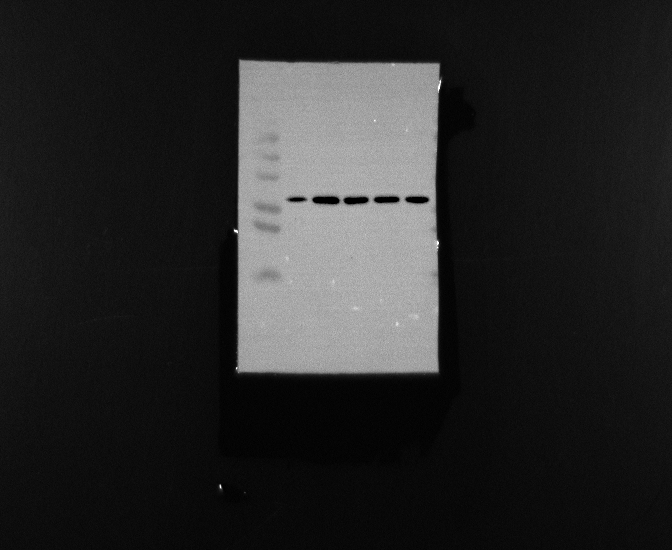

Supplement: Supplementary file 1 [file Datasheet1.zip › Date sheet 1/western blot/Figure 4G/caspase3-1.jpg]

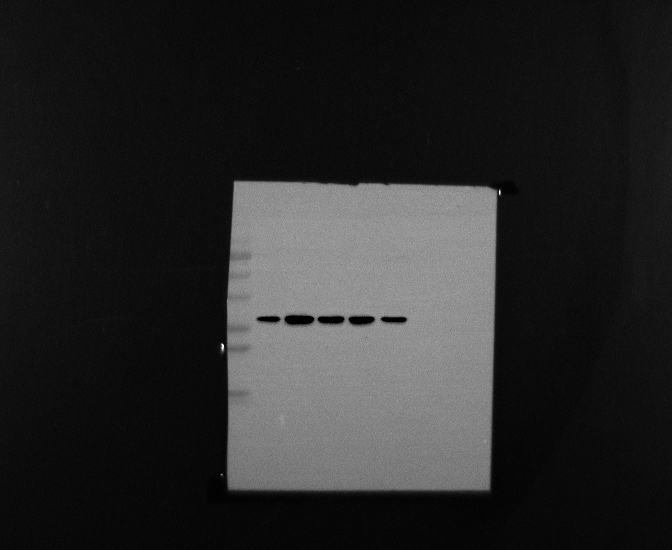

Supplement: Supplementary file 1 [file Datasheet1.zip › Date sheet 1/western blot/Figure 4G/caspase3-2.jpg]

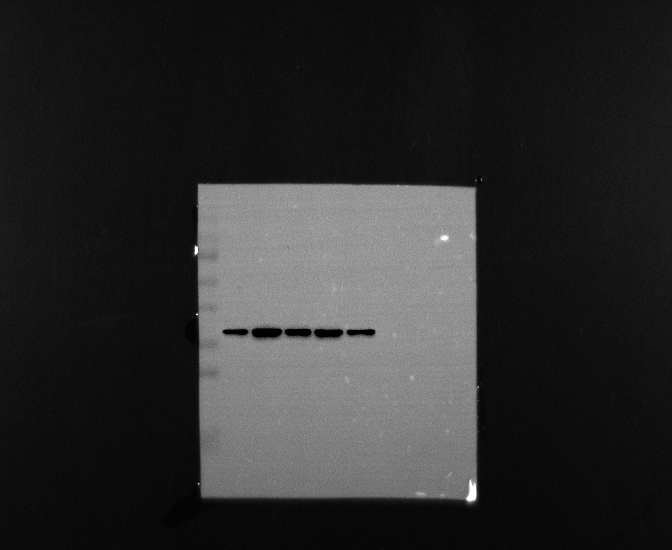

Supplement: Supplementary file 1 [file Datasheet1.zip › Date sheet 1/western blot/Figure 4G/caspase3-3.jpg]

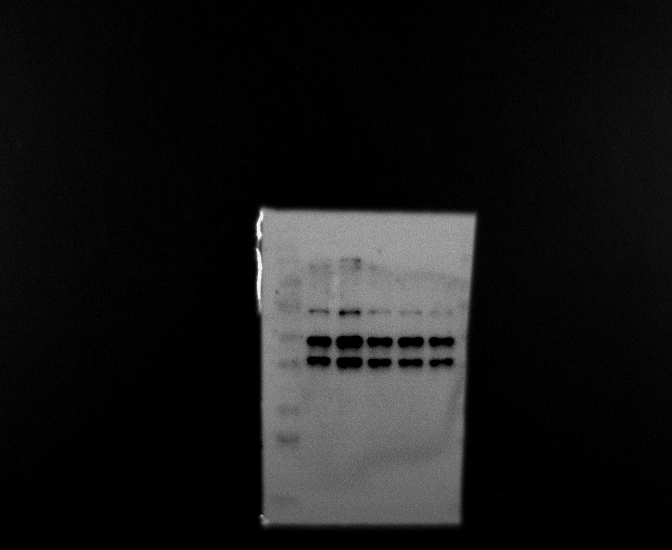

Supplement: Supplementary file 1 [file Datasheet1.zip › Date sheet 1/western blot/Figure 4G/JNK-1.jpg]

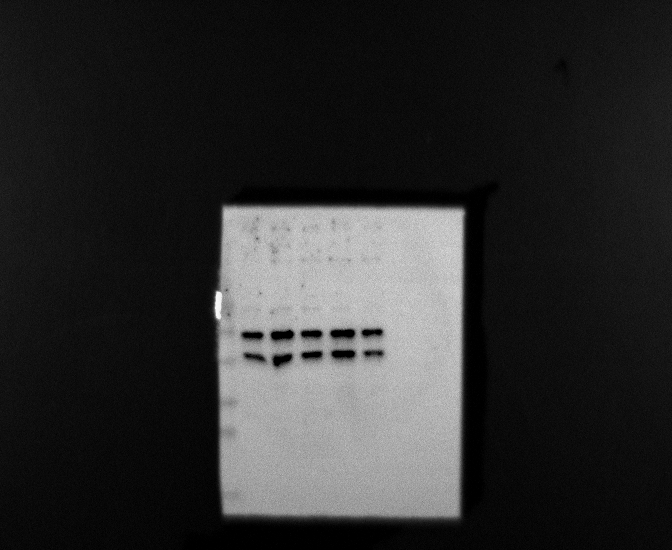

Supplement: Supplementary file 1 [file Datasheet1.zip › Date sheet 1/western blot/Figure 4G/JNK-2.jpg]

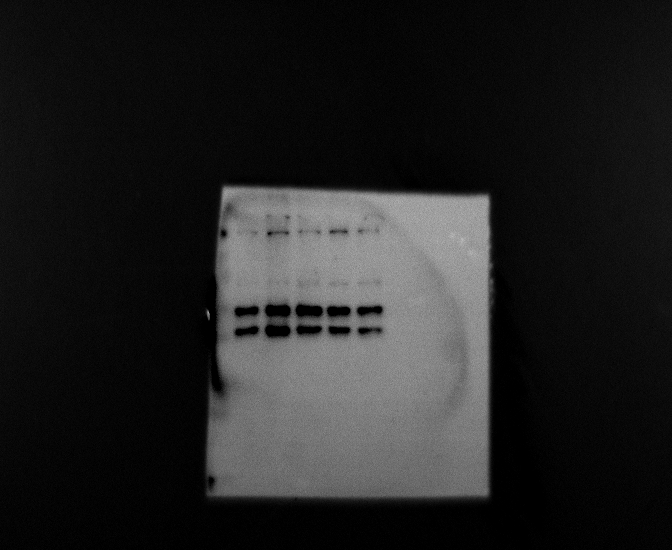

Supplement: Supplementary file 1 [file Datasheet1.zip › Date sheet 1/western blot/Figure 4G/JNK-3.jpg]

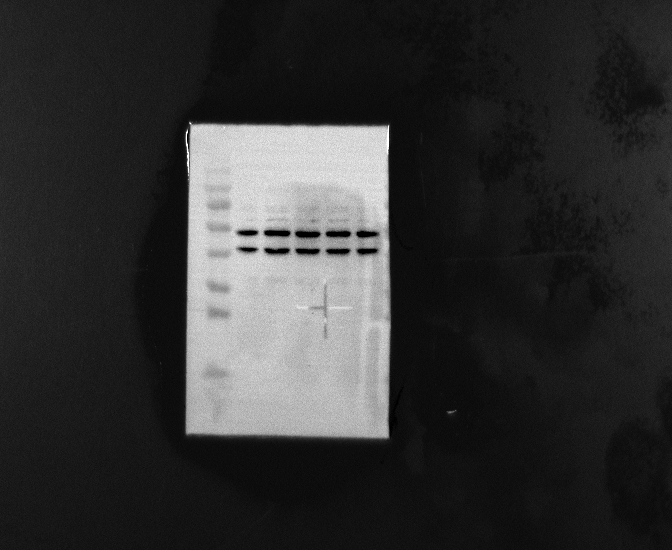

Supplement: Supplementary file 1 [file Datasheet1.zip › Date sheet 1/western blot/Figure 4G/p—JNK-1.jpg]

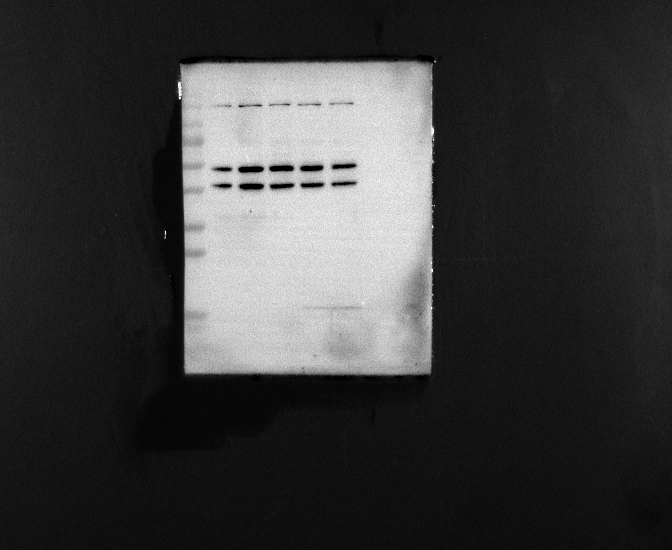

Supplement: Supplementary file 1 [file Datasheet1.zip › Date sheet 1/western blot/Figure 4G/p—JNK-2.jpg]

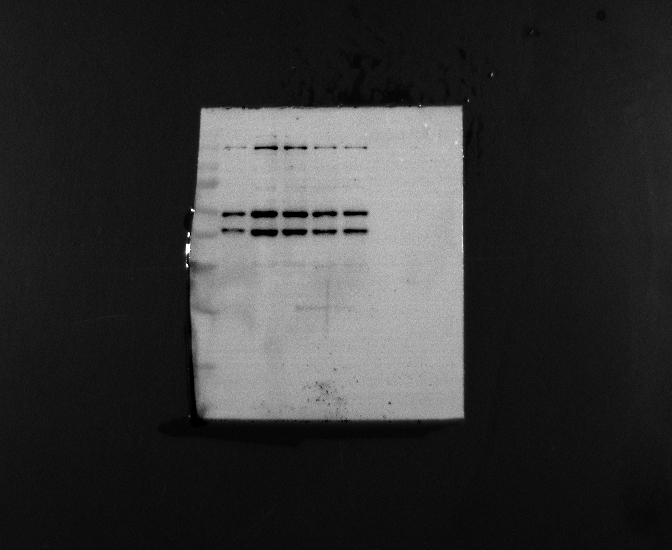

Supplement: Supplementary file 1 [file Datasheet1.zip › Date sheet 1/western blot/Figure 4G/p—JNK-3.jpg]

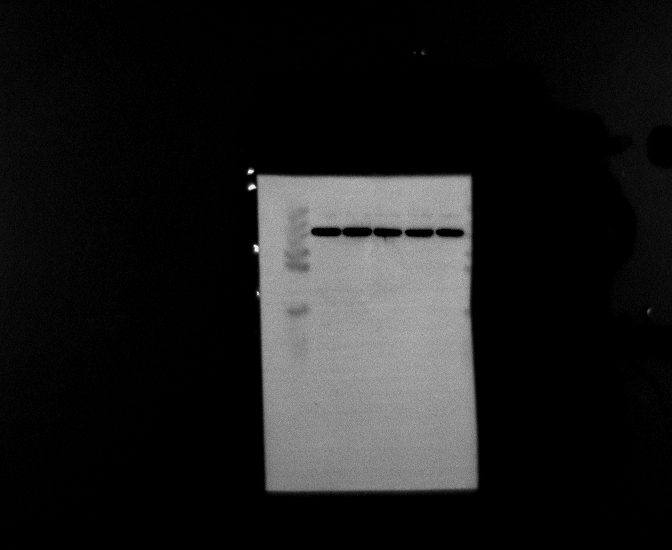

Supplement: Supplementary file 1 [file Datasheet1.zip › Date sheet 1/western blot/Figure 4G/β-actin-1.jpg]

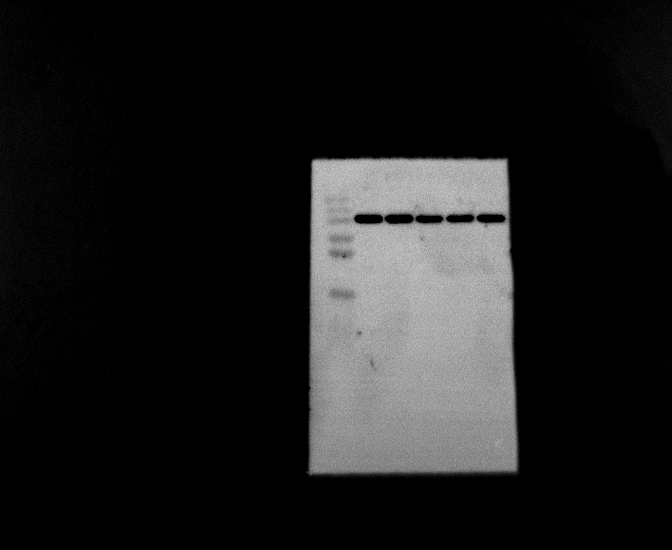

Supplement: Supplementary file 1 [file Datasheet1.zip › Date sheet 1/western blot/Figure 4G/β-actin-2.jpg]

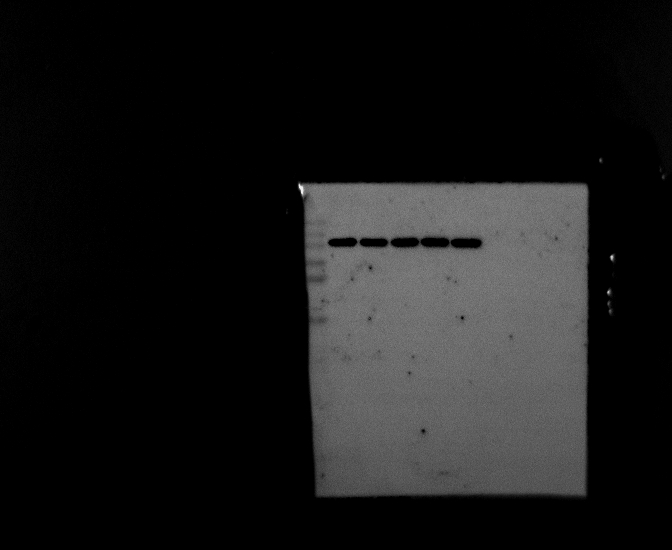

Supplement: Supplementary file 1 [file Datasheet1.zip › Date sheet 1/western blot/Figure 4G/β-actin-3.jpg]
