# Supplementary material for: Mechanism of Ershen Zhenwu Decoction in ameliorating chronic heart failure via JNK/MAPK-regulated apoptosis: insights from network pharmacology and experimental validation
Source: Front Cardiovasc Med. 2025 Apr 22;12:1561963. doi: 10.3389/fcvm.2025.1561963 (PMC12052711; doi:10.3389/fcvm.2025.1561963)
Supplement: Supplementary file 2 [file Datasheet2.pdf]

Mechanism of Ershen Zhenwu Decoction in Ameliorating Chronic Heart Failure via JNK/MAPK-Regulated Apoptosis: Insights from Network Pharmacology and Experimental Validation

Yulong Liu[1,2,#], Xinyue Wang[1,2,#], Maomao Zhang[1,2], Dan Cheng[1,2], Zhenpeng Zhu[1], Lan Ge[1,*], Xiaoyu Cheng[1,*]

[1]First Affiliated Hospital of Anhui University of Chinese Medicine, Anhui University of Chinese Medicine, Hefei, China

[2] School of Pharmacy, Anhui University of Chinese Medicine, Hefei, China

[#]Co-first authors.

***Corresponding Author:**

gelan311@126.com (Lan Ge)

cxy478@163.com (Xiaoyu Cheng)

1 Supplementary Table 1

Table 1 the 34 in *vivo* components of ESZWD.

NO.	In *vivo* ingredients	Molecular formula	Source
1	Paeoniflorin	$C_{23}H_{28}O_{11}$	Paeonia lactiflora Pall
2	Phlorizin	$C_{21}H_{18}O_{11}$	Paeonia

			lactiflora Pall
3	galloylpaeoniflorin	$C_{30}H_{33}O_{15}$	Paeonia lactiflora Pall
4	Phenylalanine	$C_9H_{11}NO_2$	Atractylodes macrocephala Koidz
5	Songorine	$C_{22}H_{31}NO_3$	Atractylodes macrocephala Koidz
6	Lecithin	$C_{42}H_{81}NO_8P$	Atractylodes macrocephala Koidz
7	1-(4-Hydroxy-3-methoxyphenyl)-3-oxo-5-decanesulfonic acid	$C_{17}H_{26}O_6S$	Salvia miltiorrhiza Bunge
8	Tanshinone IIB	$C_{19}H_{18}O_4$	Salvia

			miltiorrhiza Bunge
9	Tanshinone I	$C_{19}H_{18}O_3$	Salvia miltiorrhiza Bunge
10	Danshenxinkun B	$C_{18}H_{16}O_3$	Salvia miltiorrhiza Bunge
11	Cryptotanshinone	$C_{19}H_{20}O_3$	Salvia miltiorrhiza Bunge
12	(3E)-3-[2-[(1R,4aS,5R,6R,8aS)-6-hydroxy-5-(hydroxymethyl)-5,8a-dimethyl-2-methylidene-3,4,4a,6,7,8-hexahydro-1H-naphthalen-1-yl]ethylidene]oxolan-2-one	$C_{20}H_{30}O_4$	Salvia miltiorrhiza Bunge
13	Dimethyl D-malate	$C_6H_{10}O_5$	Poria cocos (Schw.) Wolf
14	fatsicarpain A	$C_{30}H_{46}O_4$	Poria cocos

			(Schw.) Wolf
15	Pregnanetriol	$C_{21}H_{34}O_4$	Poria cocos (Schw.) Wolf
16	Ferruginol	$C_{20}H_{30}O$	Poria cocos (Schw.) Wolf
17	Talatisamine	$C_{24}H_{39}NO_5$	Aconitum carmichaelii Debeaux
18	2,7-Dideacetyl-2,7-dibenzoyl-taxayunnanine F	$C_{42}H_{48}O_{14}$	Aconitum carmichaelii Debeaux
19	14-Ethyl-4,6,19-trimethoxy-16-methyl-9,11-dioxa-14-azaheptacyclo[10.7.2.12,5.01,13.03,8.08,12.016,20]docosane-2,21-diol	$C_{25}H_{39}NO_7$	Aconitum carmichaelii Debeaux
20	Aconitine	$C_{34}H_{47}NO_{11}$	Aconitum carmichaelii

			Debeaux
21	Acetylaconitine	$C_{36}H_{49}NO_{12}$	Aconitum carmichaelii Debeaux
22	[(2R,3R,5S,6S,8S,17R)-11-ethyl-5,8-dihydroxy-6,16-dimethoxy-11-azahexacyclo[7.7.2.12,5.01,10.03,8.013,17]nonadecan-4-yl] benzoate	$C_{29}H_{39}NO_6$	Aconitum carmichaelii Debeaux
23	8-(Acetyloxy)-20-ethyl-13-hydroxy-1,6,16-trimethoxy-4-(methoxymethyl)aconitan-14-yl benzoate	$C_{34}H_{47}NO_9$	Aconitum carmichaelii Debeaux
24	Aconine	$C_{25}H_{41}NO_9$	Aconitum carmichaelii Debeaux
25	Ginsenoside Rg1	$C_{42}H_{72}O_{14}$	Panax ginseng C.A.Mey
26	Ginsenoside Re	$C_{48}H_{82}O_{18}$	Panax ginseng C.A.Mey

27	Ginsenoside Rb1	$C_{54}H_{92}O_{23}$	Panax ginseng C.A.Mey
28	Quinquenoside R1	$C_{56}H_{94}O_{24}$	Panax ginseng C.A.Mey
29	Ginsenoside Rc	$C_{53}H_{90}O_{22}$	Panax ginseng C.A.Mey
30	Ginsenoside-Rg5	$C_{41}H_{68}O_{12}$	Panax ginseng C.A.Mey
31	Ganoderenic Acid D	$C_{31}H_{44}O_{8}$	Panax ginseng C.A.Mey
32	beta-Pinene	$C_{10}H_{16}$	Panax ginseng C.A.Mey
33	Gingerglycolipid A	$C_{33}H_{60}O_{14}$	Zingiber officinale Roscoe
34	Kaempferol 3,7-diglucoside	$C_{27}H_{29}O_{16}$	Zingiber

officinale

Roscoe

Supplementary Figure 1

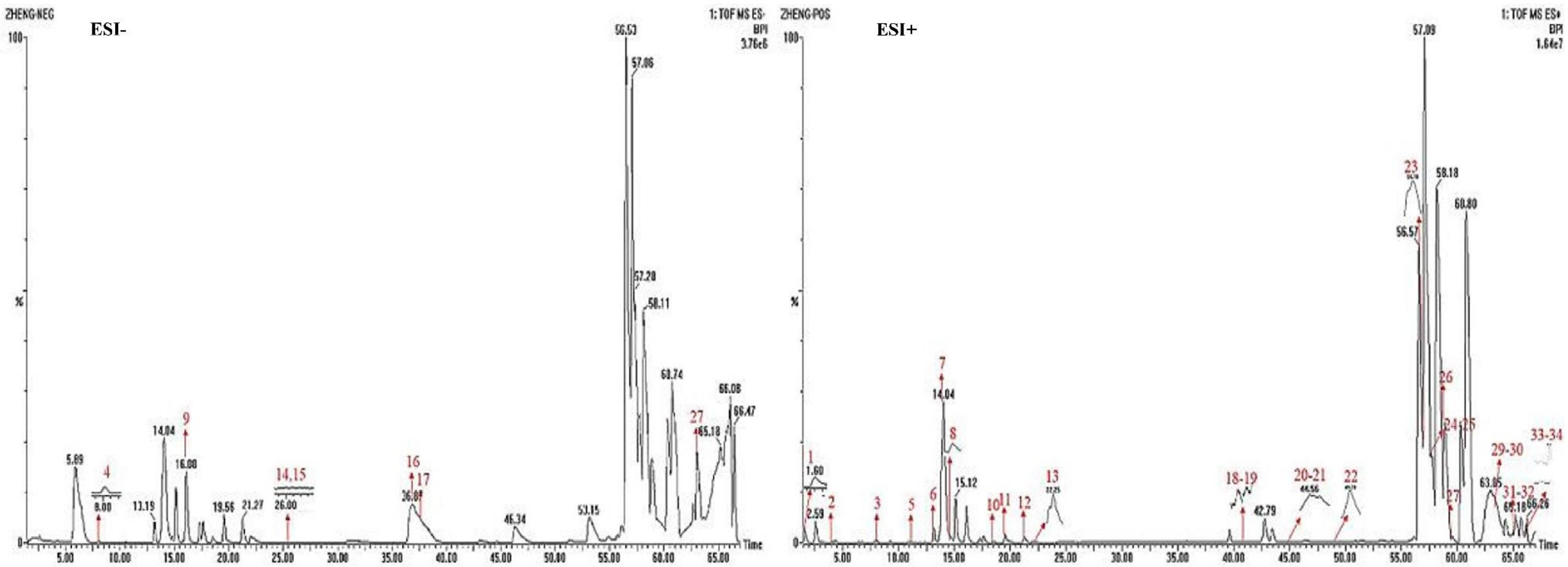

BPI chromatograms of ESZWD chemical components in normal rats under positive ion (A) and negative ion (B) modes
